# Distribution of oral and maxillofacial lesions in children and adolescents in a southeastern Brazilian population: A retrospective study including the COVID-19 pandemic period

**DOI:** 10.4317/medoral.28139

**Published:** 2026-06-28

**Authors:** Isadora Koepp Darella, João Paulo Gonçalves de Paiva, Alan Roger Santos-Silva, Jacks Jorge, Márcio Ajudarte Lopes, Pablo Agustin Vargas

**Affiliations:** 1 Departamento de Diagnóstico Oral, Faculdade de Odontologia de Piracicaba (FOP), Universidade Estadual de Campinas (UNICAMP), Piracicaba, São Paulo, Brazil

**Keywords:** Oral lesions, children, adolescents, epidemiological study, Brazil.

## Abstract

**Background:**

This retrospective study analyzed the frequency of oral and maxillofacial lesions in a pediatric population from an oral pathology service in southeastern Brazil (2015-2024).

**Materials and Methods:**

We reviewed all histopathological reports from this period, which included the COVID-19 pandemic. Demographic and clinicopathological data were collected and summarized using descriptive statistics. Associations were assessed using Pearson’s chi-squared, Fisher’s exact, Cramer’s V, and multinomial logistic regression tests.

**Results:**

Among 1,578 pediatric biopsies (patients aged 0-19 years), which represented 8.78% of the service's total caseload, adolescents were the predominant group (76.68%), with a balanced gender distribution. A significant decrease in pediatric cases was observed following the COVID-19 pandemic, although the most frequent lesion types remained consistent. Of the 1,414 conclusive diagnoses, salivary gland pathologies were most common (31.12%), followed by odontogenic cysts (18.74%) and mucosal pathologies (12.02%). Mucocele was the single most prevalent lesion (25.67%), followed by dentigerous cyst (8.17%). Statistical analysis revealed a significant association between patient age and diagnostic category, indicating distinct, developmentally dependent patterns.

**Conclusions:**

Our findings confirm the predominance of benign, reactive, and cystic lesions in pediatric patients, including during the COVID-19 pandemic, underscoring the value of current epidemiological data to guide clinical practice and preventive strategies.

## Introduction

Approximately 72 different types of lesions are associated with children and adolescents [1]. It is estimated that 7-15% of pathologies in the maxillofacial complex occur in patients within the first two decades of life [2-7]. Despite this considerable prevalence, the literature scarcely presents epidemiological studies in this population segment. Furthermore, the existing studies show significant heterogeneity regarding sample age ranges, lesion classification categories, and time periods [8].

The specific maxillofacial diseases a patient experiences are clearly connected to their developmental stage. Certain lesion groups are more common in early childhood, while others increase in incidence as patients approach adolescence and early adulthood. For these individuals, such conditions can impact on oral function, aesthetics, and mental health. These effects, including anxiety and other psychological distress for both patients and their families, can lead to long-term issues [9]. 

Given the substantial populations of both Brazil and the state of São Paulo [10], and the critical need for current epidemiological data, this study aims to analyze the distribution of oral and maxillofacial lesions diagnosed in children and adolescents (0-19 years) at a reference oral pathology center in southeastern Brazil between 2015 and 2024. The study provides temporal continuity for a previously published cohort from the same service [8]. Additionally, this study will also assess how the COVID-19 pandemic influenced the frequency of histopathological reports issued for pediatric patients.

## Materials and Methods

This study is a retrospective observational epidemiological study based on the analysis of histopathological records from an oral pathology service.

A retrospective analysis was conducted using the oral pathology records of the Oral Pathology Service of Piracicaba Dental School, State University of Campinas (FOP/UNICAMP), Piracicaba, Brazil, over a ten-year period, from January 2015 to December 2024. This study was carried out according to the ethical standards of the institutional FOP/UNICAMP research committee (protocol number 86274525.0.0000.5418) and with the 1964 Helsinki Declaration. All cases diagnosed in patients aged 19 years or younger were retrieved and compiled in a Microsoft Excel® spreadsheet.

Data on patient age at biopsy, gender, biopsy date (year), and final diagnosis were collected from the electronic system. The variables were described using absolute and relative frequencies, as well as mean (with standard deviation) and median.

Cases involving patients older than 19 years (resulting from registration errors), and recurrent cases or surgical specimens with a previous report issued by the Oral Pathology Laboratory of FOP/UNICAMP were excluded. Duplicate records (such as cases with more than one block or complementary reports) and multiple biopsies referring to the same lesion were also excluded.

To facilitate comparison with the existing literature, all lesions were classified into one of 12 categories based on the framework established by Jones *et al*. (2006) and subsequently adopted by Ha *et al*. (2014) and Ataíde *et al*. (2016). This classification was selected for its comprehensive approach to categorizing oral and maxillofacial lesions in pediatric populations. The categories were as follows: Dental pathologies, salivary gland pathologies, mucosal pathologies, odontogenic cysts, gingival/periodontal pathologies, miscellaneous pathologies, odontogenic tumors, connective tissue pathologies, bone pathologies, normal tissue, non-odontogenic cysts, malignant tumors.

The sample was stratified into two distinct age group systems. The first followed WHO criteria, dividing patients into children (0-9 years) and adolescents (10-19 years) [11]. The second system used finer groupings (0-4, 5-8, 9-12, 13-16, and 17-19 years) to assess how the distribution of lesions correlates with specific developmental stages throughout the first two decades of life [4].

Statistical analyses were performed using IBM SPSS Statistics version 22 (IBM Corporation, Armonk, NY, USA). Associations between categorical variables were assessed using Pearson's chi-square test and, when appropriate, Fisher's Exact Test.

To evaluate temporal trends across the pandemic period, cases were stratified into three timeframes: Pre-pandemic (2015-2019), pandemic (2020), and post-pandemic (2021-2024). Pairwise comparisons between these periods were conducted using Pearson's chi-square tests with calculation of odds ratios and 95% confidence intervals.

Initial bivariate analyses were conducted through contingency tables to examine associations between age class and diagnostic classification. For tables with more than 20% of cells having expected counts of less than 5, Fisher's Exact Test was preferentially employed.

The strength of associations was quantified using Cramer's V, interpreted according to established criteria: 0.10-0.30 (weak association), 0.30-0.50 (moderate association), and >0.50 (strong association). To identify specific sources of significant associations, adjusted residuals were analyzed, with absolute values exceeding 1.96 considered statistically significant.

Subsequently, multinomial logistic regression was performed to model the relationship between independent variables (gender and age class) and the dependent variable (diagnostic class), with calculation of odds ratios (OR) and 95% confidence intervals. Model fit was assessed using likelihood ratio tests and Nagelkerke pseudo R². The regression was fitted as a single global model including all diagnostic categories simultaneously. Thus, reported associations derive from the structure of the model rather than from multiple independent hypothesis tests. Salivary gland pathologies were selected as the reference diagnostic category due to their numerical predominance in the dataset, which enhances model stability and improves the precision of parameter estimates.

Across all analyses, confidence intervals were set at 95%, and two-tailed *p-*values below 0.05 were considered statistically significant.

## Results

During the ten-year study period (2015-2024), 17,982 cases were diagnosed at the FOP/UNICAMP oral pathology service. Of these, 1,578 (8.78%) involved patients aged 0 to 19 years. Regarding demographic data, the mean age was 12.72±4.42 years, with a median of 13 years. According to the World Health Organization (WHO) age classification, 368 patients (23.32%) were children (0-9 years), while 1,210 (76.68%) were adolescents (10-19 years) (Table 1).

In the stratification of age groups based on development, the distribution of patients was as follows: 72 (4.56%) were 0-4 years old, 211 (13.37%) were 5-8, 405 (25.67%) were 9-12, 522 (33.08%) were 13-16, and 368 (23.32%) were 17-19. The gender distribution was balanced, with 795 females (50.38%) and 783 males (49.62%), resulting in a female-to-male ratio of 1.02:1 (Table 1, Figure 1).

From 2015 to 2024, the laboratory's total caseload followed a distinct triphasic pattern: A phase of progressive growth peaking at 1,949 cases in 2019, a sharp decline to 1,073 cases in 2020 coinciding with the COVID-19 pandemic, and a post-pandemic recovery that surged to a record 2,477 cases by 2024. 

Throughout this period, pediatric cases (0-19 years) represented a stable minority of the total caseload. While their absolute numbers fluctuated annually (ranging from 87 to 197), their proportional share remained within a narrow band of 6.97% to 10.51% of annual diagnoses (Figure 2).

Statistical analysis of these proportions, however, revealed a significant structural shift underlying this apparent stability (Table 2). 

The immediate decline in the pediatric share from 9.7% pre-2020 to 8.1% in 2020 was not statistically significant (*p=*0.103), suggesting the pandemic's initial impact was proportionally similar across pediatric and adult patients. Furthermore, no significant difference was found between the pandemic (8.1%) and post-pandemic (8.0%) periods (*p=*0.872), indicating a stabilization at this new, lower level.

The critical finding emerged from the direct comparison of the pre- and post-pandemic eras. This analysis demonstrated a highly significant reduction in the proportional representation of pediatric cases, from 9.7% to 8.0% (*p<*0.001). An odds ratio of 1.234 (95% CI: 1.109-1.374) quantified this shift, revealing that the odds of a case being pediatric were 23.4% higher in the pre-pandemic period.

This evidence points to a lasting alteration in the laboratory's case profile, indicating that the post-pandemic recovery in overall volume has not proportionally reinstated pediatric diagnostic activity (Figure 2). Notably, despite this shift in caseload composition, the distribution of the most frequent diagnoses remained consistent between the pre-pandemic and post-pandemic periods Supplementary 1 Table S1 (https://www.medicinaoral.com/medoralfree01/28139/Suppl1.pdf).

Of the 1,578 reports issued by the laboratory during the study period, 1,414 (89.61%) provided a conclusive diagnosis, while the remaining 164 (10.39%) were released as descriptive findings Supplementary 1 Table S2 (https://www.medicinaoral.com/medoralfree01/28139/Suppl1.pdf).

The full data regarding the frequency of each diagnostic category is presented in the Supplementary 1 S3-S14 (https://www.medicinaoral.com/medoralfree01/28139/Suppl1.pdf).

All lesions with a conclusive diagnosis were classified into one of the twelve established categories. Among the 1,414 cases analyzed, salivary gland pathologies were the most prevalent, comprising 440 cases (31.12%). This was followed by odontogenic cysts (n=265; 18.74%) and mucosal pathologies (n=170; 12.02%). Gingival and periodontal pathologies accounted for 10.33% (n=146), while odontogenic tumors represented 8.84% (n=125). The remaining categories each constituted less than 8% of the total, including dental pathologies (n=104; 7.36%), bone pathologies (n=52; 3.68%), non-odontogenic cysts (n=43; 3.04%), and connective tissue pathologies (n=40; 2.83%). The least frequent findings were miscellaneous pathologies (n=17; 1.20%), malignant tumors (n=10; 0.71%), and normal tissues (n=2; 0.14%). Collectively, these results underscore a clear predominance of benign and inflammatory conditions, particularly those affecting the salivary glands and of odontogenic origin Supplementary 1 Table S2 (https://www.medicinaoral.com/medoralfree01/28139/Suppl1.pdf).

The mucous extravasation phenomenon (mucocele) was the most frequently diagnosed lesion, with 405 cases (25.67%). It was followed by dentigerous cysts (n=129; 8.17%) and fibrous hyperplasia (n=88; 5.58%). Other notable diagnoses included normal dental follicles (n=78; 4.94%), odontoma (n=71; 4.50%), pyogenic granuloma (n=60; 3.80%), odontogenic keratocyst (n=60; 3.80%), and squamous papilloma (n=44 cases; 2.79%). Less common entities, each representing less than 2% of cases, were the solitary bone cyst (n=31; 1.96%), ameloblastoma (n=29; 1.84%), central giant cell granuloma (n=24; 1.52%), ranula (n=22; 1.39%), and peripheral ossifying fibroma (n=16; 1.01%). Collectively, the fifteen most prevalent lesions accounted for 73.26% of all diagnoses, underscoring that while a wide variety of pathologies may present in this age group, the clinical workload is dominated by a relatively short list of common conditions Supplementary 1 Table S15,S16 (https://www.medicinaoral.com/medoralfree01/28139/Suppl1.pdf).

The distribution of the most frequent diagnoses within each pathological category is detailed in Supplementary 1 Table S3-S14 (https://www.medicinaoral.com/medoralfree01/28139/Suppl1.pdf), with their respective frequency in the total sample shown in Supplementary 1 Table S16 (https://www.medicinaoral.com/medoralfree01/28139/Suppl1.pdf). The normal dental follicle was the predominant dental pathology, comprising 75% of its category (Supplementary 1 Table S3 (https://www.medicinaoral.com/medoralfree01/28139/Suppl1.pdf) and representing 4.94% of all specimens (n=78; Supplementary 1 Table S16 (https://www.medicinaoral.com/medoralfree01/28139/Suppl1.pdf). Within salivary gland pathologies, the mucous extravasation phenomenon (mucocele) was dominant, accounting for 92.05% of this group (Supplementary 1 Table S4(https://www.medicinaoral.com/medoralfree01/28139/Suppl1.pdf) and 25.67% of the total sample (n=405; Supplementary 1 Table S16 (https://www.medicinaoral.com/medoralfree01/28139/Suppl1.pdf). For mucosal pathologies, fibrous hyperplasia was the most prevalent finding, representing 51.76% of its category (Supplementary 1 Table S5 (https://www.medicinaoral.com/medoralfree01/28139/Suppl1.pdf) and 5.58% of all cases (n=88; Supplementary 1 Table S16 (https://www.medicinaoral.com/medoralfree01/28139/Suppl1.pdf).

Among odontogenic cysts, the dentigerous cyst was the most common, constituting 48.68% of these cysts (Supplementary 1 Table S6 (https://www.medicinaoral.com/medoralfree01/28139/Suppl1.pdf) and 8.17% of all diagnoses (n=129; Supplementary 1 Table S16 (https://www.medicinaoral.com/medoralfree01/28139/Suppl1.pdf). In gingival and periodontal pathologies, pyogenic granuloma was the most frequent, comprising 41.10% of this category (Supplementary 1 Table S7 (https://www.medicinaoral.com/medoralfree01/28139/Suppl1.pdf) and 3.80% of all specimens (n=60; Supplementary 1 Table S16 (https://www.medicinaoral.com/medoralfree01/28139/Suppl1.pdf). The most prevalent finding among miscellaneous pathologies was nonspecific ulceration, accounting for 41.18% of the group (Supplementary 1 Table S8 (https://www.medicinaoral.com/medoralfree01/28139/Suppl1.pdf) and 0.44% of the total (n=7; Supplementary 1 Table S16 (https://www.medicinaoral.com/medoralfree01/28139/Suppl1.pdf).

Odontoma was the predominant odontogenic tumor, representing 56.80% of these lesions (Supplementary 1 Table S9 (https://www.medicinaoral.com/medoralfree01/28139/Suppl1.pdf) and 4.50% of all diagnoses (n=71; Supplementary 1 Table S16 (https://www.medicinaoral.com/medoralfree01/28139/Suppl1.pdf), with the compound odontoma being its most frequent variant (70.42% of odontomas; Supplementary 1 Table S10 (https://www.medicinaoral.com/medoralfree01/28139/Suppl1.pdf). In connective tissue pathologies, myofibroma, lymphangioma, and capillary hemangioma were equally prevalent, with each representing 12.5% of the group (Supplementary 1 Table S11 (https://www.medicinaoral.com/medoralfree01/28139/Suppl1.pdf) and 0.32% of the total sample (each n=5; Supplementary 1 Table S16 (https://www.medicinaoral.com/medoralfree01/28139/Suppl1.pdf). The central giant cell granuloma was the most frequent bone pathology, comprising 46.15% of its category (Supplementary 1 Table S12 (https://www.medicinaoral.com/medoralfree01/28139/Suppl1.pdf) and 1.52% of all cases (n=24; Supplementary 1 Table S16 (https://www.medicinaoral.com/medoralfree01/28139/Suppl1.pdf). The solitary bone cyst was the predominant non-odontogenic cyst, accounting for 72.09% of these cysts (Supplementary 1 Table S13 (https://www.medicinaoral.com/medoralfree01/28139/Suppl1.pdf) and 1.96% of all diagnoses (n=31; Supplementary 1 Table S16 (https://www.medicinaoral.com/medoralfree01/28139/Suppl1.pdf). Finally, among malignant tumors, Langerhans cell histiocytosis and metastatic neuroblastoma were the most common entities, each constituting 20% of this group (Supplementary 1 Table S14 (https://www.medicinaoral.com/medoralfree01/28139/Suppl1.pdf)) and 0.13% of the total sample (each n=2; Supplementary 1 Table S16 (https://www.medicinaoral.com/medoralfree01/28139/Suppl1.pdf). The most prevalent lesions in each class are illustrated in Figure 3.

A statistically significant association was observed between age group and pathological classification (Fisher's Exact Test, *p<*0.001). The analysis revealed distinct developmental patterns: Malignant tumors were most prevalent in the youngest cohort (0-4 years), salivary gland pathologies peaked in early childhood (5-8 years), while dental pathologies and cystic lesions became increasingly common throughout adolescence. A Cramer's V value of 0.185 indicated a weak-to-moderate strength of association, confirming that age is a relevant, though not overwhelmingly dominant, factor in the distribution of these lesions (Table 3).

Multinomial logistic regression analysis identified significant age-dependent patterns in oral diagnostic classifications, accounting for approximately 12% of the diagnostic variability (Nagelkerke R²=0.12). The model revealed several pronounced associations: connective tissue pathologies showed substantially higher odds in early childhood (OR = 7.228; 95% CI: 1.950-26.794; *p=*0.003), while the odds of dental and odontogenic cystic pathologies decreased progressively from infancy through early adolescence. A similar protective effect was observed for non-odontogenic cysts (OR=0.165; 95% CI: 0.036-0.757; *p=*0.020) and odontogenic tumors (OR=0.264; 95% CI: 0.115-0.605; *p=*0.002) in the 5-8-year-old age group. Furthermore, the odds of gingival and periodontal pathologies peaked significantly during adolescence (OR=1.770; 95% CI: 1.004-3.122; *p=*0.048 for the 13-16 year group) (Table 4).

An analysis of the descriptive reports revealed that the primary reason for an inconclusive diagnosis was insufficient, inadequate, or unrepresentative biopsy material, which accounted for most cases (n=132; 80.49%). The second most common reason was a lack of essential clinical and/or radiographic information (n=22; 13.41%). A small proportion of cases (n=10; 6.10%) were hindered by a combination of both inadequate material and missing complementary data (Supplementary 1 Table S17 (https://www.medicinaoral.com/medoralfree01/28139/Suppl1.pdf).

## Discussion

This study provides one of the most comprehensive epidemiological analyses of oral and maxillofacial lesions in a Brazilian pediatric population, offering crucial insights into age-dependent diagnostic patterns and the impact of the COVID-19 pandemic on oral pathology services.

The overall proportion of pediatric cases in our sample (8.78%) aligns with the 7-15% range reported in the literature, confirming that children and adolescents represent a consistent minority of oral biopsy specimens across different geographic regions and age classifications [2-7]. The demographic profile of our cohort, characterized by a predominance of adolescents (76.68%) and a balanced gender distribution, further corroborates well-established epidemiological trends [11]. This adolescent predominance is driven by greater exposure to risk behaviors, key developmental changes, and clinical practices that often defer intervention due to the challenges of performing invasive procedures in younger children [4,5,8,9,11-14]. While the data confirm a general increase in oral pathology frequency from childhood to adolescence [4,8], a notable reduction in the absolute number of cases was observed in the 17-19-year-old group. However, the proportional distribution of diagnostic categories remained stable between the older adolescent groups, suggesting this decrease may be more closely related to changes in healthcare-seeking behavior, such as decreased dental attendance with increasing autonomy, rather than a true biological decline in disease incidence.

While dental caries is the most prevalent oral disease in young populations [15], our findings robustly confirm that other maxillofacial lesions are not uncommon [5]. The profile of pediatric oral lesions is dominated by benign conditions. Although the overall occurrence of many maxillofacial lesions in children is low [8], reactive lesions such as mucocele and fibrous hyperplasia are common in this patient group [8,9,11]. In pre-adolescents and adolescents, cysts and benign tumors, particularly those of odontogenic origin, become more frequent than in younger children, a pattern recognized in the literature [11,16]. This is reflected in our results, with salivary gland pathologies being the most frequent diagnostic group (31.12%), primarily mucocele (25.67% of all diagnoses), and odontogenic cysts (18.74%), led by the dentigerous cyst (8.17%).

This pattern is highly consistent with other Brazilian studies. A comparative analysis with a previous study from our service [8] reveals a stable predominance of salivary gland and mucosal pathologies, although with slight proportional differences between the two cohorts (salivary gland pathologies: 37.1% in 2000-2014 vs. 31.12% in the current series; mucosal pathologies: 13.6% vs. 12.02%). In the present study, the most frequent diagnostic categories were salivary gland pathologies, odontogenic cysts, mucosal pathologies, gingival and periodontal diseases, and odontogenic tumors, whereas in the previous cohort the order was salivary gland pathologies, mucosal pathologies, odontogenic cysts, gingival and periodontal diseases, and dental pathologies. Notably, our study showed a proportional increase in odontogenic cysts and tumors (18.74% and 8.84%, respectively), contributing to a shift in the relative ranking of diagnostic categories over time. These findings reinforce the overall stability of the diagnostic profile while suggesting subtle temporal variations in specific lesion groups.

Furthermore, when compared to other Brazilian regions [1,5], mucocele consistently emerges as the single most common lesion, followed by a similar spectrum of reactive, cystic, and odontogenic conditions, indicating a homogeneous national epidemiological profile. These findings can be explained by high susceptibility to trauma and the prolonged period of tooth exfoliation and eruption, which predisposes individuals to mucoceles and dentigerous cysts, respectively, particularly from the second decade of life [8,9,17,18]. 

It is critical to acknowledge that this biopsy-based profile is inherently influenced by clinical practice, as many common entities such as dental pathologies and odontomas are often managed without histopathological confirmation [8]. Consequently, this design introduces an inherent selection bias, as the analyzed sample primarily reflects lesions that were clinically indicated for biopsy rather than the full spectrum of pediatric oral conditions. As a result, the relative frequencies reported here may not fully represent the true epidemiological distribution of oral pathologies in the pediatric population. Therefore, the findings should be interpreted within the context of biopsy-based diagnostic services and caution should be exercised when extrapolating these results to the broader population.

The multinomial logistic regression model significantly associated age with diagnostic category, revealing distinct patterns that mirror key developmental stages. The peak of salivary gland lesions in children aged 5-8 years aligns with increased exposure to local trauma [18], while the rise in dental and odontogenic cystic pathologies in adolescents corresponds to permanent tooth eruption, cumulative inflammatory insults, and the time required for developmental lesions to manifest [19]. The peak of gingival pathologies in adolescents (13-16 years) further coincides with pubertal hormonal changes [14]. Although the strength of this association was moderate (Cramer's V=0.185), it robustly confirms age as a crucial determinant of disease presentation, a trend also highlighted by previous Brazilian and international studies [4,5,8].

At the other end of the spectrum, malignant neoplasms were confirmed to be exceedingly rare (0.71% of cases), reinforcing the established profile of pediatric oral pathology [8,9,13]. The specific frequency of Langerhans cell histiocytosis and metastatic neuroblastoma in our cohort, while diverging from some reports, underscores that different malignancies can predominate in specific age groups and geographic settings [13,20,21]. Despite their rarity, these conditions demand vigilant clinical attention due to their potential severity.

Although the regression model was statistically significant, its modest explanatory power (Nagelkerke R²=0.12) indicates that age and gender account for only a limited proportion of the observed diagnostic variability. This finding is expected in multifactorial clinical settings, where lesion distribution reflects the interaction of biological development, environmental exposure, healthcare access, and referral practices. Variables not captured in this retrospective dataset, such as anatomical site, clinical presentation, referral source, and socioeconomic background, may substantially influence diagnostic patterns. Therefore, the model should be interpreted as identifying meaningful age-dependent trends rather than fully explaining the determinants of lesion distribution.

Our analysis suggests that the pandemic period was associated with a temporary reduction in case volume, which may reflect changes in healthcare-seeking behavior and service availability rather than a direct causal effect on underlying disease patterns. This interpretation is supported by the absence of a specific association between SARS-CoV-2 infection and oral lesions in our pediatric cohort, contrary to reports in the general population [22]. It is further supported by a comprehensive systematic review that identified temporal availability factors, such as the closure of non-urgent services and prolonged waiting times, as well as psychological factors, notably fear of contracting the virus in clinical settings, as potential barriers that may have disrupted care pathways during this period [23].

However, the most relevant finding was the persistent reduction in the proportion of pediatric cases in the post-pandemic period. Although statistically significant, this difference should be interpreted cautiously, as the absolute variation was modest. Importantly, this study did not directly assess healthcare access variables, socioeconomic indicators, referral patterns, institutional service capacity, or demographic changes in the population served. Therefore, causal inferences regarding permanent structural changes in the healthcare system cannot be established. While pandemic-related disruptions in healthcare pathways, driven by temporary service restrictions and psychological barriers to care-seeking, may have contributed to this pattern [23], other explanations should also be considered. These may include delayed presentation of pediatric patients, changes in referral dynamics, differential recovery between adult and pediatric services, or even shifts in the demographic profile of the population served by the institution. Given the absence of comparable pediatric oral pathology studies, direct comparisons remain limited, and the observed reduction should be interpreted as a hypothesis-generating finding that warrants further investigation.

Although the overall distribution of lesions resembles patterns previously reported in pediatric oral pathology, several aspects differentiate the present study. This analysis covered a ten-year period (2015-2024) and included a large sample derived from a major referral center, with a high annual diagnostic volume (mean of 1,798 cases per year). A standardized classification of lesions was applied, and complementary statistical analyses were performed using two age stratifications-one based on WHO criteria and another reflecting developmental stages. Additionally, this study provides, to our knowledge, the first assessment of the impact of the COVID-19 pandemic on the diagnostic profile of pediatric oral lesions and allows comparison with a previous cohort from the same research group to explore temporal changes. Continuous epidemiological studies remain essential, as patterns of oral lesions may vary across populations due to demographic, behavioral, and healthcare access factors, and updated data are important to document both emerging trends and the persistence of established patterns.

## Conclusions

This study delineates key epidemiological patterns in pediatric oral pathology. The 0-19 age group accounted for a substantial proportion of cases, with adolescents predominating over children due to a combination of developmental, behavioral, and clinical factors. We observed a predictable evolution in diagnostic profiles with advancing age, yet the overall landscape remains overwhelmingly characterized by benign reactive and cystic lesions. Notably, the pandemic period was associated with a transient reduction in case volume without distorting these fundamental epidemiological trends. Consistent with established evidence, malignant and aggressive pathologies were confirmed to be distinctly rare in the pediatric population. 

## Figures and Tables

**Table 1:** Table 1: Demographic data of pediatric patients included in this study.

**Variable**	**n (%)**
Demographics	1,578 (100)
Age	
*Age range (years)*	0-19
*Mean years (SD)*	12.72 (4.42)
*Median years*	13
WHO classification	
*0 to 9 years*	368 (23.32)
*10 to 19 years*	1,210 (76.68)
Development-based age groups	
*0 to 4 years*	72 (4.56)
*5 to 8 years*	211 (13.37)
*9 to 12 years*	405 (25.67)
*13 to 16 years*	522 (33.08)
*17 to 19 years*	368 (23.32)
Gender	
*Female*	795 (50.38)
*Male*	783 (49.62)
*Female:Male ratio*	1.02:1

n: Number. SD: Standard deviation. WHO: World Health Organization.

**Table 2:** Table 2: Comparative analysis of the proportion of pediatric reports between pre-pandemic, pandemic, and post-pandemic periods.

**Period**	**Proportion of pediatric reports (%)**	*p* **-value**	**Odds ratio (CI 95%)**
Pre-pandemic vs. Pandemic	9,7% vs. 8,1%	0,103	1,211 (0,961-1,525)
Pandemic vs. Post-pandemic	8,1% vs. 8,0%	0,872	1,019 (0,807-1,287)
Pre-pandemic vs. Post-pandemic	9,7% vs. 8,0%	<0,001	1,234 (1,109-1,374)

CI: Confidence interval.

**Table 3:** Table 3: Association between age class and diagnostic class.

**Age Class**	**1**	**2**	**3**	**4**	**5**	**6**	**7**	**8**	**9**	**10**	**11**	**12**	**Total**	*p* **-value**
0-4 years (n=67)														<0.001^ab^
*Count*	1	23	13	2	9	2	1	7	1	0	3	5	67	
*% within Age*	1.5%	34.3%	19.4%	3.0%	13.4%	3.0%	1.5%	10.4%	1.5%	0.0%	4.5%	7.5%	100%	
*Adj. Residual*	-1.8*	0.5	1.7	-3.0*	0.8	1.3	-2.0*	3.7*	-0.9	-0.3	0.7	6.6*		
5-8 years (n=197)														
*Count*	7	96	28	16	19	6	8	6	7	0	2	2	197	
*% within Age*	3.6%	48.7%	14.2%	8.1%	9.6%	3.0%	4.1%	3.0%	3.6%	0.0%	1.0%	1.0%	100%	
*Adj. Residual*	-2.0*	4.4*	0.9	-3.4*	-0.3	2.4*	-2.3*	0.2	-0.1	-0.5	-1.6	0.5		
9-12 years (n=366)														
*Count*	16	114	48	75	47	3	36	9	13	1	2	2	366	
*% within Age*	4.4%	31.1%	13.1%	20.5%	12.8%	0.8%	9.8%	2.5%	3.6%	0.3%	0.5%	0.5%	100%	
*Adj. Residual*	-2.1*	0.0	0.6	0.8	1.5	-0.7	0.6	-0.4	-0.1	0.7	-2.7*	-0.4		
13-16 years (n=461)														
*Count*	44	112	45	100	48	4	50	14	19	0	24	1	461	
*% within Age*	9.5%	24.3%	9.8%	21.7%	10.4%	0.9%	10.8%	3.0%	4.1%	0.0%	5.2%	0.2%	100%	
*Adj. Residual*	1.7*	-2.6*	-1.4	1.5	0.1	-0.7	1.4	0.3	0.5	-0.8	2.7*	-1.3		
17-19 years (n=323)														
*Count*	36	95	36	72	23	2	30	4	12	1	12	0	323	
*% within Age*	11.1%	29.4%	11.1%	22.3%	7.1%	0.6%	9.3%	1.2%	3.7%	0.3%	3.7%	0.0%	100%	
*Adj. Residual*	2.5*	-0.5	-0.5	1.5	-1.8*	-1.0	0.3	-1.7	0.0	0.8	0.7	-1.5		
Total	104	440	170	265	146	17	125	40	52	2	43	10	1414	
% Overall	7.4%	31.1%	12.0%	18.7%	10.3%	1.2%	8.8%	2.8%	3.7%	0.1%	3.0%	0.7%	100%	

Adj. Residual: Adjusted standardized residual, %:

**Table 4:** Table 4: Multinomial logistic regression analysis between age class and diagnostic class.

**Diagnostic Class**	**Age Group**	**OR**	**95% CI**	*p-* **value**
Dental pathologies	0-4 years	0.115	0.015-0.881	0.037
	5-8 years	0.192	0.082-0.454	<0.001
	9-12 years	0.370	0.194-0.709	0.003
	13-16 years	1.037	0.617-1.741	0.892
	17-19 years	Ref	-	-
Odontogenic cysts	0-4 years	0.115	0.026-0.503	0.004
	5-8 years	0.220	0.119-0.405	<0.001
	9-12 years	0.868	0.569-1.325	0.512
	13-16 years	1.178	0.783-1.772	0.431
	17-19 years	Ref	-	-
Gingival/periodontal pathologies	0-4 years	1.616	0.660-3.956	0.293
	5-8 years	0.817	0.418-1.598	0.556
	9-12 years	1.703	0.965-3.006	0.066
	13-16 years	1.770	1.004-3.122	0.048
	17-19 years	Ref	-	-
Connective tissue pathologies	0-4 years	7.228	1.950-26.794	0.003
	5-8 years	1.484	0.406-5.428	0.550
	9-12 years	1.875	0.560-6.281	0.308
	13-16 years	2.969	0.945-9.323	0.062
	17-19 years	Ref	-	-
Non-odontogenic cysts	0-4 years	1.033	0.269-3.962	0.963
	5-8 years	0.165	0.036-0.757	0.020
	9-12 years	0.139	0.030-0.636	0.011
	13-16 years	1.696	0.805-3.573	0.164
	17-19 years	Ref	-	-
Odontogenic tumors	0-4 years	0.138	0.018-1.063	0.057
	5-8 years	0.264	0.115-0.605	0.002
	9-12 years	1.000	0.574-1.743	1.000
	13-16 years	1.414	0.833-2.399	0.199
	17-19 years	Ref	-	-

OR: Odds ratio, CI: Confidence interval, Ref: Reference. Likelihood Ratio Test: χ^2^(44)=175.294, p<0.001. Nagelkerke Pseudo R^2^=0.119. Reference Category: Salivary gland pathologies (diagnosis), 17-19 years (age).

**Figure 1:**
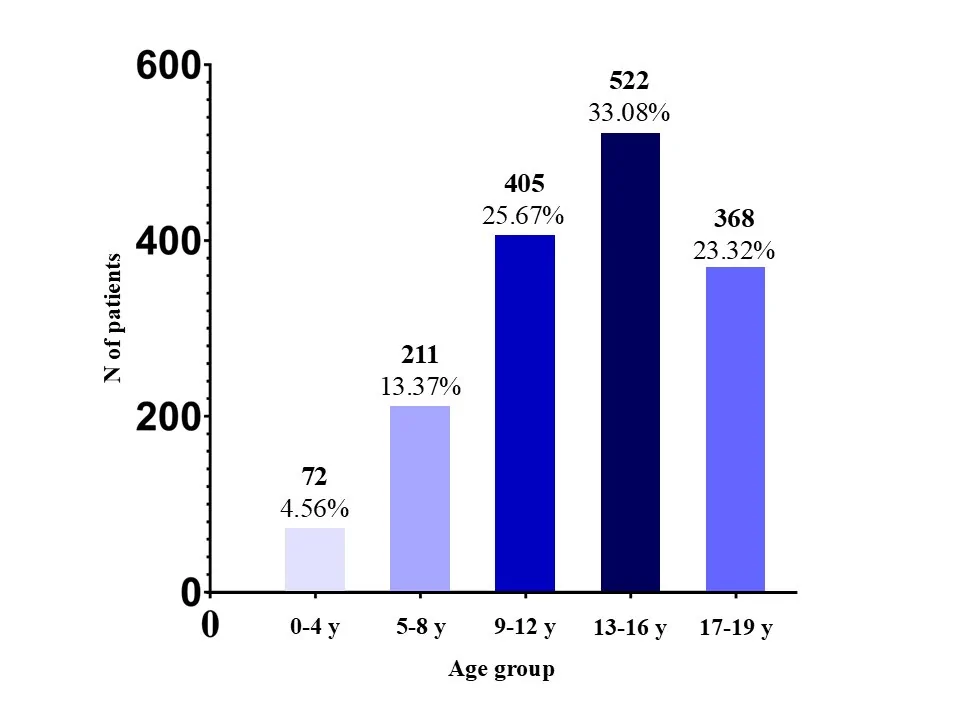
Fig. 1: Distribution of oral lesions in the studied pediatric population, according to development-based age groups.

**Figure 2:**
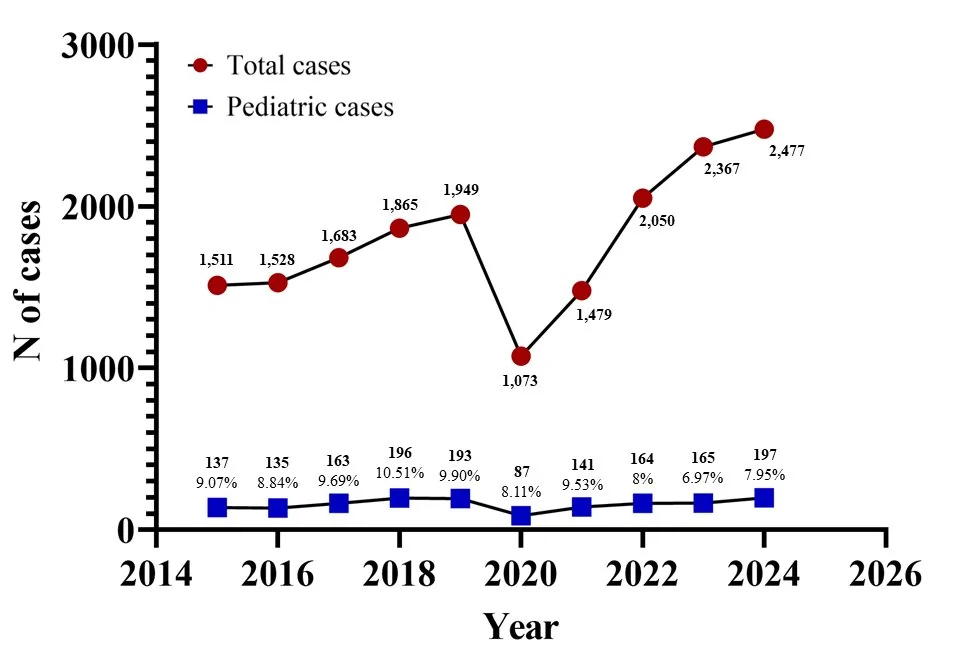
Fig. 2: Annual variation in the number of total and pediatric cases between 2014 and 2025.

**Figure 3:**
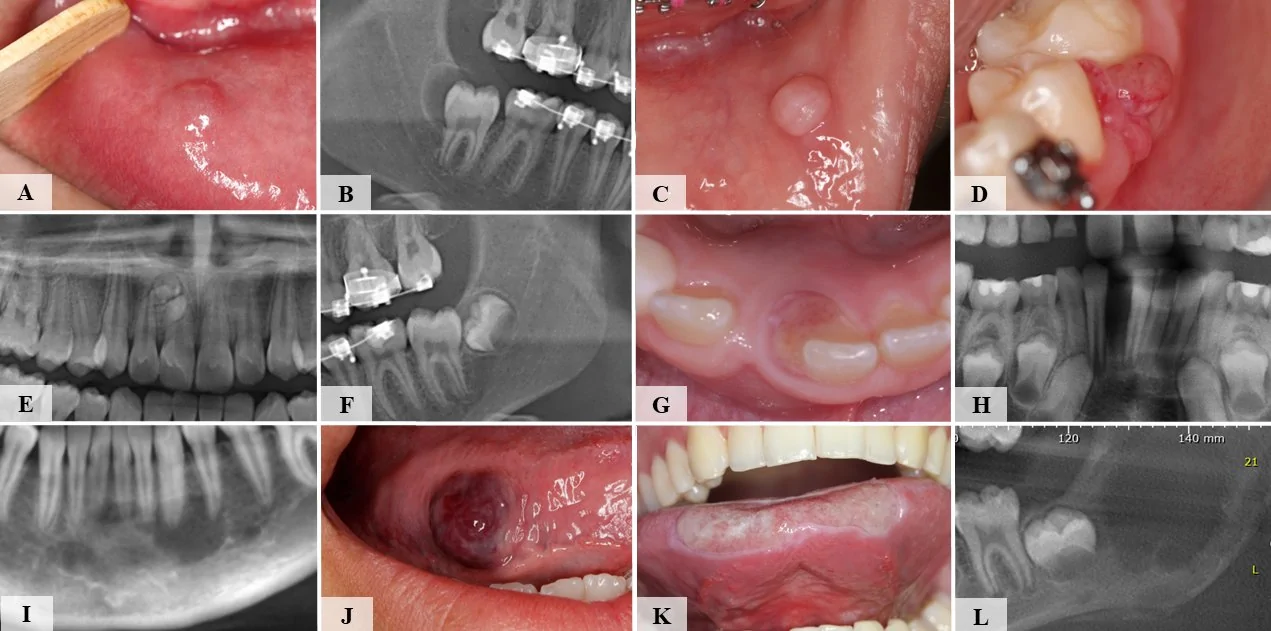
Fig. 3: Clinical and radiographical aspects of the most prevalent lesions in each diagnostic class. A) Mucocele; B) Dentigerous cyst; C) Fibrous hyperplasia; D) Pyogenic granuloma; E) Odontoma; F) Pericoronal hood/follicle; G-H) Central giant cell lesion; I) Simple bone cyst; J) Hemangioma; K) Nonspecific chronic ulceration; L) Langerhans cell histiocytosis.

## Data Availability

None.
